# Companion-Animal Abuse in South Korea: Welfare and Veterinary Implications from Criminal-Court Decisions (2003–2025)

**DOI:** 10.3390/ani16152354

**Published:** 2026-08-02

**Authors:** Ah-Young Kim, Ji-Su Baek, BokKyung Ku, Kyunghyun Lee

**Affiliations:** Animal Disease Diagnostic Division, Animal and Plant Quarantine Agency, Gimcheon 39660, Republic of Korea

**Keywords:** companion animal abuse, criminal court decisions, Animal Protection Act, sentencing, animal welfare, South Korea

## Abstract

Animal abuse directly compromises companion-animal welfare, causing pain, injury, neglect, and, in many cases, death. Although veterinary case series and welfare surveys offer clinical perspectives, far less is known about how abuse is documented in criminal-court decisions. This study analyzed 501 publicly available judgments issued under the South Korean Animal Protection Act between 2003 and 2025 to characterize the species involved, the abusive acts described, sentencing outcomes, and the visibility of veterinary information. Dogs were the predominant victims (63.9%), followed by a heterogeneous group of other or unspecified species (22.1%) and cats (14.0%). Blunt-force trauma (beating, kicking, or hitting with objects) was the most common coded act (92.0%), whereas neglect-related death (3.0%), asphyxiation (2.6%), and sharp-force trauma (2.4%) were recorded far less frequently. Most offenders received monetary fines (64.5%), with custodial sentences imposed in 14.1% of cases. Veterinary information was not consistently visible in the public legal record; a minimum of 7 of 501 decisions (1.4%) contained an unambiguous, explicit reference to veterinary involvement, and this floor estimate likely understates the true frequency, because many appellate summaries omitted the factual detail needed to determine whether veterinary records or expert opinions had been presented at earlier stages. The results therefore characterize how prosecuted abuse is represented in publicly accessible court records rather than the full spectrum of animal abuse or the severity of suffering experienced by the animals. When situated within the limited but growing international literature, the South Korean findings resemble other judgment-based datasets in foregrounding overt violence more readily than chronic or diffuse forms of welfare harm.

## Simple Summary

Animal abuse harms companion animals by inflicting pain, injury, or neglect, yet criminal-court records often provide only limited information about welfare consequences. This study examined 501 publicly available criminal-court decisions issued under the South Korean Animal Protection Act between 2003 and 2025. Most cases involved dogs and blunt-force trauma, such as beating or kicking, whereas neglect-related deaths were far less frequently described in the legal record. Courts generally imposed fines rather than imprisonment and explicitly documented veterinary information was unevenly visible across the publicly accessible judgments. These findings show how court decisions can reveal recurring patterns of prosecuted abuse while also underscoring that judicial records provide only a partial view of the broader animal-abuse burden.

## 1. Introduction

Animal abuse is a serious concern because it can cause injury, neglect, prolonged distress, and death [1,2,3]. Companion animals, particularly dogs and cats, are especially vulnerable because of their close proximity to humans and dependence on human care [1,2,3]. Veterinary medicine provides clinical and forensic tools for documenting injury and neglect, including forensic pathology and clinical case series, but many prosecuted cases are visible to researchers chiefly through legal records rather than detailed medical files [1,2,3]. Court judgments are formal records of recognized offenses and sanctions. However, they are not designed as clinical or welfare documents and often omit examination findings, necropsy results, treatment history, or behavioral observations [1,2,3]. This feature limits what can be inferred from judgments alone about the extent, duration, and physiological consequences of harm [1,2,3]. In South Korea, companion-animal abuse is prohibited under the Animal Protection Act, and public concern about animal welfare has grown alongside debate about enforcement and sentencing [4,5,6,7,8]. Previous scholarship has examined legislative development, legal limitations, and sentencing practices in Korean law, as well as broader national welfare policies and the current state of animal cruelty law [4,5,6,7,8]. Examining criminal-court decisions under that framework can clarify which animals and abusive acts are most visible in the judicial record, what circumstances are described by courts, and how often veterinary information is explicitly mentioned. Veterinarians occupy a critical position at the interface between clinical practice, welfare protection, and legal processes. Studies from South Korea and comparative contexts have examined practitioners’ intention to report suspected abuse, ethical and professional considerations around reporting, and the forensic investigation of suspected cruelty, including toxicological analysis in animal fatalities [9,10,11]. These findings highlight both the potential and the constraints of veterinary involvement when abuse is suspected, especially in systems where mandatory reporting is not yet fully standardized [9,10,11]. Internationally, empirical research on criminal-justice responses to animal abuse remains limited but is gradually expanding. Judgment-based studies from Finland, Brazil, the Czech Republic, Estonia, and the United States have shown that court records and prosecution data can reveal recurring patterns in victim species, offense types, evidentiary practices, and sanctions, while also showing how strongly such findings depend on jurisdiction, legal definitions, and data source selection [12,13,14,15,16,17,18,19]. Positioning South Korean judgments within this literature helps distinguish features that may be specific to the national legal context from patterns that recur across different legal systems [12,13,14,15,16,17,18,19].

This study therefore uses criminal-court decisions under the Animal Protection Act as a structured dataset to describe prosecuted companion-animal abuse cases and to examine what those records make visible. The study was designed as a descriptive analysis rather than a retrospective welfare severity assessment. Specifically, the aims were to:

- Characterize the animals and abusive acts recorded in judgments;
- Summarize the contextual factors described by courts;
- Present the distribution of sentencing outcomes;
- Describe whether and how veterinary information appears in the accessible judicial texts.

By treating court decisions as legally structured but welfare-relevant texts, this study highlights both the value and the limits of judicial records for animal welfare research. The findings identify documentation gaps, clarify what court records can and cannot show, and point to the importance of stronger collaboration among veterinarians, welfare researchers, investigators, and legal professionals [1,2,3,9,10,11,12,13,14,15,16,17,18,19].

## 2. Materials and Methods

### 2.1. Case Identification and Selection

Criminal-court decisions were identified through publicly accessible South Korean judicial-decision repositories, primarily the online judgment database of the Supreme Court of Korea. Searches were conducted for criminal judgments adjudicated under the Animal Protection Act between 2003 and July 2025, using keywords related to animal abuse offenses, including “animal cruelty”, “animal abuse”, and “Animal Protection Act violations”.

The search targeted criminal decisions from publicly accessible court levels available through the repository, including first-instance and appellate decisions when published. Inclusion required explicit textual descriptions of (1) at least one abusive act toward an animal, (2) the affected animal or animals, and (3) a finalized judicial outcome. If the same case appeared at multiple stages, appellate decisions were retained only once for the case and duplicate records were excluded. Consolidated cases were treated as a single case when the court decision addressed them together. Duplicate identification was based on overlapping case numbers, court level, decision dates, factual narratives, and defendant information where available. Decisions without a finalized sentencing outcome were not included. The final dataset comprised 501 criminal-court decisions that met these predefined criteria.

### 2.2. Veterinary Information in Judicial Records

For the purposes of this study, “veterinary information” was defined as any clinical record, necropsy report, diagnostic test result, treatment record, or written opinion by a licensed veterinarian that was explicitly mentioned in the judicial decision. Such information was not routinely included in the publicly accessible records, and many decisions contained no medical or forensic detail beyond a brief description of the animal’s condition or fate. We initially intended to classify each decision into one of three categories: veterinary information explicitly visible; no veterinary information visible despite a sufficiently detailed factual narrative; or not assessable because the decision was too abbreviated to determine either outcome. However, the narrative depth of the accessible judgments—particularly appellate summaries—varied too widely to apply this three-way distinction reliably and consistently across the full dataset. We therefore report, in Section 3.5, only the minimum number of decisions containing an unambiguous, explicit reference to veterinary involvement, which should be interpreted as a floor estimate rather than a complete case-by-case classification of veterinary-information visibility. Accordingly, the coding of abusive acts and case characteristics relied primarily on the judgment narratives, and veterinary documentation was recorded only when explicitly visible in the text of the accessible decision.

### 2.3. Variable Definitions and Coding Scheme

The unit of analysis was the individual criminal-court decision. From each decision, information was coded into predefined categories. The species of the affected animal or animals was recorded as dogs, cats, or other species, with the latter category including birds, livestock, reptiles, rodents, mixed-species cases, and appellate decisions in which the species was not specified.

Abusive acts were coded according to the principal act emphasized in the judgment narrative. The categories were blunt-force trauma (e.g., beating, kicking, or hitting with objects), sharp-force trauma (e.g., cutting or stabbing), asphyxiation-related acts (e.g., strangulation or hanging), and neglect-related death (e.g., prolonged deprivation of food, water, shelter, or care leading to death). These labels should be understood as operational coding categories for legal texts rather than as validated forensic-severity classes. When multiple forms of abuse were described in a single case, coding prioritized the act that appeared to constitute the main harmful conduct in the narrative or the act most central to the charged offense. Because the available legal texts often summarized events briefly, the coding framework was designed to capture the main described form of abuse rather than every act within a case.

The resulting categories mix mechanisms of injury and a neglect-based fatal outcome. This approach was retained because the court narratives themselves often described violence by mechanism but neglect by prolonged omission and resultant death rather than by a single discrete injury mechanism. The categories therefore function as pragmatic descriptors of how abusive conduct was represented in the legal texts, not as a unified pathophysiological taxonomy.

Basic defendant characteristics, such as sex, were recorded when explicitly reported. Reported contextual factors surrounding the abusive act were coded only when they appeared in the court’s descriptions—for example, references to noise, indoor defecation, escape attempts, handling failures, or interactions with stray animals. These factors were recorded strictly as reported circumstances rather than inferred motivations.

Sentencing outcomes were coded based on the sanctions reported in the judgment, including fines only, fines combined with probation, and imprisonment with or without suspension, as well as whether concurrent offenses were applied. Veterinary information was coded as visible when the decision explicitly mentioned clinical records, necropsy findings, diagnostic results, treatment records, or written expert opinions, and as not visible when no such references appeared in the accessible text.

Two coders independently coded an initial set of 50 decisions using the predefined coding manual. Inter-coder agreement for species and abusive-act category was substantial (Cohen’s kappa = 0.82 and 0.72, respectively), and discrepancies were resolved through discussion, leading to minor refinements of the category definitions. After this pilot, one coder completed the remaining coding, with periodic checks of randomly selected cases by the second coder.

### 2.4. Data Completeness and Selection Constraints

Information on some variables was incomplete or absent in a proportion of decisions. Defendant sex and detailed contextual factors were not specified in many records, and veterinary information could not always be assessed because some publicly accessible judgments, especially appellate summaries, omitted the factual detail needed to determine whether such evidence had been presented at trial. In addition, the dataset included only publicly available court decisions with sufficient narrative detail and a finalized judicial outcome. It therefore represents a selected subset of prosecuted cases rather than the full population of animal abuse events, reported complaints, filed prosecutions, or all adjudicated cases in South Korea. The findings should be interpreted as describing the visible end of the criminal process as represented in publicly accessible records.

### 2.5. Analytical Approach

The analysis was descriptive and retrospective. Frequencies and proportions were calculated for species, categories of abusive acts, contextual factors, and sentencing outcomes. Narrative summaries were used to describe recurring patterns in how abusive acts and judicial responses were represented in the decisions. No inferential statistical testing or formal severity scoring was performed, because standardized clinical assessments and comprehensive veterinary documentation were not consistently available from the legal texts. The study did not attempt to retrospectively grade suffering severity, duration of distress, pain intensity, or the proportionality of sentences on the basis of judgment narratives alone.

## 3. Results

### 3.1. General Characteristics of the Cases

A total of 501 criminal-court decisions involving animal abuse were included in this analysis (Table 1).

Dogs were the most frequently recorded species, accounting for 63.9% (320/501) of cases, followed by cats at 14.0% (70/501). Other species comprised 22.1% (111/501) of the dataset. Male defendants were identified in 40.7% (204/501) of cases and female defendants in 4.2% (21/501), whereas defendant sex was not specified in 55.1% (276/501) of the judicial records.

### 3.2. Categories of Abusive Acts in Judicial Narratives

The coding of abusive acts based on the judgment narratives is summarized in Table 2.

Blunt-force acts were the predominant coded category, observed in 92.0% (461/501) of cases. Neglect-related death accounted for 3.0% (15/501) of cases. Asphyxiation-related acts were identified in 2.6% (13/501) of cases, while sharp-force trauma was reported in 2.4% (12/501).

### 3.3. Contextual Factors Reported in Decisions

When courts provided contextual information about the circumstances of abusive acts, the most commonly recorded category involved reported nuisance-related circumstances, such as excessive barking or indoor defecation, described in the narrative immediately before the act. In other cases, narratives referred to difficulties in controlling or restraining animals, including escape attempts or handling failures, and to encounters with stray animals entering private property. These factors are summarized in Table 3.

In 25.9% (130/501) of cases, no clear contextual factor could be identified from the judgment text because such details were not described. Across the dataset, information on prior living conditions, behavioral history, and long-term outcomes was generally sparse.

### 3.4. Sentencing Outcomes

Sentencing outcomes are summarized in Table 4.

The three sentence-type categories were mutually exclusive and sum to 501 (100%). Concurrent offenses were recorded in 58 cases (11.6%) as a separate case-level attribute overlapping these categories. The majority of decisions resulted in fines only (64.5%, 323/501). Fines combined with probation were imposed in 21.4% (107/501) of cases, and imprisonment, including suspended sentences, was observed in 14.1% (71/501) of cases.

### 3.5. Visibility of Veterinary Information in Judicial Records

Explicit references to veterinary information—including clinical records, necropsy reports, diagnostic findings, treatment records, or expert opinions—were not consistently visible in the decisions reviewed. A reliable case-by-case classification distinguishing decisions with no veterinary information from decisions in which this could not be assessed was not possible for the full dataset, because many records, particularly appellate summaries, omitted the factual material needed to make this distinction with confidence. As a minimum-bound estimate, an unambiguous, explicit reference to veterinary involvement (e.g., veterinary diagnosis prior to euthanasia, veterinarian-performed euthanasia, or unlicensed medical procedures performed on an animal) was identified in 7 of 501 decisions (1.4%). The true frequency of veterinary information in the underlying judgments is very likely higher than this figure, because more detailed forensic or clinical evidence (such as necropsy findings) discussed at trial may not have been carried into the abbreviated case narratives reviewed for this study, especially in appellate summaries. Accordingly, veterinary information is best characterized as inconsistently and only partially visible in the accessible texts, rather than quantified as a reliable prevalence estimate.

## 4. Discussion

This study analyzed 501 publicly accessible criminal-court decisions under the South Korean Animal Protection Act to describe how prosecuted companion-animal abuse is represented in judicial records. Dogs were the most frequently recorded victims, blunt-force trauma was the dominant coded category, and fines were more common than custodial sentences. Veterinary information was unevenly visible in the accessible judgments, limiting what could be concluded from the legal record alone [1,2,3,5,6]. The findings should be interpreted in light of the study design and source material. Because the dataset includes only publicly available decisions with sufficient narrative content and finalized judicial outcomes, it represents a selective subset of prosecuted abuse cases rather than the totality of abuse incidents or even the full universe of criminal proceedings [3,4,7,8,12,13,14,15,16,17,18,19]. This selection process introduces substantial risk of bias. Cases that were never reported, not prosecuted, resolved without publication, or published only in highly abbreviated form are outside the dataset, and those missing cases may differ systematically in species, abuse pattern, severity, evidentiary profile, or sentencing outcome. The present results therefore describe patterns in visible court records, not the epidemiology of animal abuse in South Korea.

Placed in an international context, the overall species pattern is broadly consistent with prior studies of prosecuted companion-animal abuse, although the relevant comparison set is narrower than the full body of international literature cited in this manuscript. In Massachusetts, Arluke and Luke reported that dogs were the most common victims and that dogs and cats together accounted for the vast majority of prosecuted physical-cruelty incidents [18]. Likewise, Finnish judgment-based research on companion-animal welfare offences by Valtonen and colleagues found that dogs were the most frequently targeted companion animals, followed by cats and other species, while also documenting a substantial burden of passive neglect and large-scale welfare offenses [13]. By contrast, Finnish farm-animal enforcement data concern cattle and pig welfare violations on farms and are not a directly comparable dataset with respect to companion-animal species prevalence, even though they originate from the same national judicial system [12]. These parallels among companion-animal judgment datasets [13,18,19] suggest that criminal-justice records in different jurisdictions often foreground harm to familiar companion species, while the exact mix of offenses depends strongly on legal and institutional context.

The predominance of blunt-force trauma in the present material should be interpreted cautiously. The data show that blunt force was the most frequently coded category in the available judgments, but the dataset does not directly test why this pattern appears. One possible explanation is that overt physical violence is more readily described, evidenced, or prosecuted than chronic neglect or diffuse welfare compromise, as suggested by veterinary forensic reviews and companion-animal judgment-based work in other jurisdictions [1,2,3,13,18]. However, this remains an interpretation rather than a direct empirical finding of the present study. What can be stated with confidence is that the public legal record in this dataset captured blunt-force descriptions far more often than neglect-related death, sharp-force trauma, or asphyxiation-related acts. Comparison with Finnish companion-animal data is informative here: Valtonen and colleagues reported a much greater prominence of passive neglect in prosecuted companion-animal welfare offences, including failures involving feeding, care, housing, and veterinary treatment [13]. The difference between that pattern and the present South Korean dataset may reflect real jurisdictional variation, but it may also reflect differences in inspection systems, prosecution practices, publication patterns, and the legal treatment of chronic neglect [3,4,7,13,18]. Accordingly, the current findings should not be taken to mean that blunt-force trauma is inherently the dominant form of companion-animal abuse in South Korea overall; rather, it is the dominant form in this selected set of publicly accessible judgments.

The contextual factors described in the South Korean decisions—such as nuisance-related circumstances, handling difficulties, or stray-animal encounters—also require cautious interpretation. Because judgments often provide limited narrative detail, these categories should not be treated as verified motives or psychological explanations. They are best understood as circumstances recorded in the accessible texts immediately surrounding the abusive act, consistent with methodological cautions raised in prior Korean legal scholarship [4,7]. At the same time, studies examining how defendants and courts frame the circumstances of animal-welfare offences in other jurisdictions offer relevant, if not directly comparable, context: Finnish research on the neutralization techniques used by defendants charged with animal welfare offences shows how offenders rationalize the circumstances surrounding their conduct [16]; Estonian scholarship on the “mental suffering” paradigm in judicial and media treatment of animal cruelty illustrates how courts and media frame the seriousness of abuse [17]; and judgment- or prosecution-based studies from Brazil, the Czech Republic, and the United States similarly show that judicial narratives can reflect broader societal attitudes toward animals beyond the bare facts of the offense [14,15,18,19]. Sentencing patterns in the present study also resemble international observations that non-custodial sanctions frequently predominate in animal abuse cases. In the Massachusetts dataset analyzed by Arluke and Luke, fines and probation were common and jail sentences occurred in only a minority of cases [18]. Finnish and Czech judgment-based work likewise indicates that financial penalties and suspended sentences are often favored over custodial sanctions in animal welfare offences, although the details vary by legal system and, in the Finnish case, between companion-animal and farm-animal welfare enforcement [12,13,15]. Korean scholarship has highlighted similar tendencies toward fines and relatively limited use of imprisonment in animal cruelty cases under national law [4,5,7]. The South Korean data therefore fit within a broader pattern in which criminal courts often respond to animal abuse with monetary penalties or suspended sanctions [3,4,5,7,12,13,15,18,19]. However, the present study cannot determine whether any particular sentence was proportionate to animal suffering because the judgments did not provide sufficiently standardized or comprehensive welfare evidence for such an assessment [1,2,3,11,17].

The visibility of veterinary information remains an important but constrained aspect of the present dataset. The study objective was not to estimate the true prevalence of veterinary involvement across all prosecuted cases, but rather to describe whether and how veterinary evidence was visible in publicly accessible judicial texts [1,2,3,9,10,11]. Because the accessible records frequently omitted the factual detail needed to determine whether clinical or expert evidence had been presented, a full three-way classification of visibility could not be applied reliably across the dataset; the study instead reports a minimum-bound count of decisions containing an unambiguous, explicit veterinary reference (7/501, 1.4%), which almost certainly underestimates the true frequency of veterinary involvement in the underlying cases [1,2,3,9,10,11,17]. Prior work on veterinarians’ reporting intentions, ethical roles, and forensic contributions underscores the importance of clearer pathways for integrating clinical and forensic evidence into legal processes when abuse is suspected [1,2,3,9,10,11].

### Limitations

Several limitations should be considered when interpreting these findings. First, the analysis was restricted to publicly accessible criminal decisions with finalized judicial outcomes and sufficient narrative detail; unreported abuse, non-prosecuted cases, unpublished cases, and minimally described decisions were not represented. Second, this selection process creates substantial risk of bias, because the visible court record may differ systematically from the full population of abuse cases. Third, court judgments are not designed as welfare documents, and the depth of factual description varied considerably, particularly in appellate summaries. Fourth, veterinary information could not be quantified reliably from the accessible texts because factual summaries were often incomplete. Fifth, abusive acts were classified according to the principal act described in the narrative; cases involving multiple or overlapping forms of abuse may therefore have been simplified. Finally, the study was descriptive and retrospective: it did not grade suffering severity, pain intensity, duration of compromise, or sentence proportionality, and no inferential statistical testing was performed.

## 5. Conclusions

This study shows how publicly accessible criminal-court decisions under the South Korean Animal Protection Act represent prosecuted companion-animal abuse. In this selected set of 501 judgments, dogs were the most frequently recorded victims, blunt-force trauma was the most common coded abusive act, and fines were more common than custodial sanctions. Neglect-related death and other non-blunt-force categories appeared less often in the accessible legal record.

These findings should be interpreted as characteristics of the published judicial record rather than as direct estimates of the full prevalence, severity, or welfare burden of companion-animal abuse in South Korea. The judgments reviewed did not provide a sufficient basis for reliable assessment of suffering severity, duration, or sentence proportionality. The study therefore supports cautious conclusions about visibility and documentation, not definitive conclusions about the full welfare impact of abuse across all cases.

The limited and inconsistent visibility of veterinary information in published judgments further indicates that the legal record often captures only part of the evidentiary picture. Stronger integration of veterinary documentation into judicial processes may improve how harm is recorded and understood, but future work should also address the broader selection limits of court-based datasets.

## Figures and Tables

**Table 1:** Characteristics of animal abuse cases included in this study (N = 501).

Variable	*n* (%)
**Species**	
Dogs	320 (63.9)
Cats	70 (14.0)
Other species *	111 (22.1)
**Defendant sex**	
Male	204 (40.7)
Female	21 (4.2)
Unknown/not specified	276 (55.1)

* Other species include birds, livestock, reptiles, rodents, mixed-species cases, as well as appellate cases listed without specific species information.

**Table 2:** Categorization of abusive acts based on adapted forensic descriptors.

Abuse Category	Operational Definition	Cases (*n*, %)
Blunt-force trauma	Beating, kicking, or hitting with objects	461 (92.0)
Sharp-force trauma	Cutting or stabbing injuries	12 (2.4)
Asphyxiation-related acts	Strangulation or hanging	13 (2.6)
Neglect-related death	Prolonged deprivation of food, water, shelter, or care leading to death	15 (3.0)

**Table 3:** Contextual factors described in judicial narratives.

Reported Contextual Factor	Description	Cases (*n*, %)
Reported nuisance-related circumstance	Noise, defecation, disobedience	214 (42.7)
Animal handling difficulty	Escape attempts, handling failure	96 (19.2)
Stray-animal encounter	Stray-animal intrusion	61 (12.2)
Unspecified	Not described in court records	130 (25.9)

**Table 4:** Judicial outcomes of animal abuse cases (N = 501).

Sentence Type	Cases (*n*, %)
Fine only	323 (64.5)
Fine with probation	107 (21.4)
Imprisonment (including with probation)	71 (14.1)

## Data Availability

The data presented in this study are available within the manuscript.
